# Chemical eye injuries: a 10 year retrospective review of acute presentations and clinical outcomes in Auckland, New Zealand

**DOI:** 10.1038/s41598-024-58670-y

**Published:** 2024-04-09

**Authors:** Natalie E. Allen, Alexandra Z. Crawford, Charles N. J. McGhee, Jay J. Meyer

**Affiliations:** Department of Ophthalmology, The University of Auckland, Auckland, New Zealand

**Keywords:** Health care, Risk factors, Medical research, Epidemiology, Outcomes research

## Abstract

To assess the aetiologies, clinical characteristics, treatment regimens, and outcomes of acute chemical injuries treated at an emergency eye clinic. Retrospective, observational study of all cases of chemical eye injury that presented acutely to the Greenlane Clinical Centre in Auckland, New Zealand from 1 January 2012 through 31 December 2021. Patient demographics, activity at the time of injury, causative chemical, clinical characteristics of injury at presentation, severity (Dua) classification, admission and discharge best corrected visual acuity (BCVA), treatment regimen, time to epithelisation and number of follow-up appointments were recorded. In total, 1522 cases involving 1919 eyes were studied. The mean age was 40.6 ± 18.8 years and 65% were male. The majority of cases occurred at home (62%) and cleaning was the most common activity (38%). There were 1490 Grade I (98%), 22 Grade II (1.5%), 5 Grade III (0.3%), 1 Grade IV (0.07%), 0 Grade V, and 4 Grade VI (0.3%) cases. An epithelial defect was noted in 409 cases (26.9%), of which re-epithelialisation occurred within one week for 378 cases (92%) and within 30 days for 384 cases (94%). Moderate vision loss (BCVA ≤ 6/12) attributed to the injury occurred in 152 (10%), while severe vision loss (BCVA ≤ 6/60) occurred in 30 (2%). Lack of irrigation at the scene was associated with an increased risk of severe injury and longstanding visual impairment (*p* = 0.0001). Most acute chemical injuries are mild with good clinical outcomes. Although rare, severe injuries are associated with a lack of irrigation at the scene and worse visual outcomes.

## Introduction

Chemical eye injuries are considered an ophthalmologic emergency due to the risk of significant ocular morbidity or even blindness. Worldwide, the annual incidence of ocular chemical injury is between 0.02 and 50 per 100,000 with an estimated 107,000 people being blinded by chemical injury every year^1–6^. While young male industrial workers have traditionally been considered the highest-risk population, there is evidence that young children in a domestic setting are emerging as a vulnerable patient group^7,8^.

Despite the frequency and potential severity of chemical eye injuries, there is limited literature on the demographic features, medical management and outcomes of these patients. The purpose of this study was to characterise the patient demographics, clinical characteristics, management, and outcomes of ocular chemical injuries in a developed country with a national public health service with free access at the point of care: New Zealand—Aotearoa.

## Methods

This was a retrospective study of all cases that presented with acute chemical eye injuries to a major, public teaching hospital, Greenlane Clinical Centre, in Auckland, New Zealand, over a 10-year period from 1 January 2012 through 31 December 2021. Greenlane Clinical Centre is the primary referral centre for ophthalmic emergencies in the greater Auckland Metropolitan area, catering to a population of approximately 1.7 million people. It is the only public emergency eye clinic for this catchment population. The study received approval from the Auckland Health Research Ethics Committee (AH23363). This research complies with the Declaration of Helsinki for research involving human participants and was conducted in accordance with all guidelines and regulations outlined by the University of Auckland. Exclusion criteria included thermal injury only and eyes referred for tertiary subspecialty care who had already received acute treatment at another hospital.

Patients were identified through electronic searches of diagnosis coding and searches of key terms through the Auckland clinical records department. Data collection was performed by reviewing the electronic medical record of each patient.

Data recorded included patient demographics, the setting where the injury occurred, the nature of the chemical injury, the severity of the injury (Dua Classification)^9^, treatment including the need for surgical interventions, baseline best corrected visual acuity (BCVA, Snellen), and intraocular pressure (IOP). Outcome measures included time to reepithelialisation and final BCVA. The number of eyes with moderate or severe vision loss was recorded. Moderate visual loss was defined as best-corrected visual acuity (BCVA) ≤ 6/12 and severe vision loss was vision ≤ 6/60. Statistical analysis was performed using Microsoft Excel 2013. Rates of moderate to severe injury and rates of longstanding visual impairment post-injury, with and without prompt irrigation, at the scene were examined with a two-tailed Chi-square test. A *p*-value of < 0.05 was considered statistically significant.

## Results

A total of 1776 cases were initially identified as potential chemical injuries by electronic searches for diagnoses that included pertinent keywords and terms. Upon review of each chart, 68 (3.82%) were excluded due to being purely thermal burns and 186 (10.5%) were excluded for being alternate diagnoses, leaving a total of N = 1522 patients (1919 eyes) for analysis. In 397 patients, both eyes were injured. In the case of bilateral involvement, only the most severe eye was included for analysis.

The mean number of follow-up appointments was 0.55 with the highest number of follow-up appointments for any one patient being 66. Patients were considered lost to follow-up if they had a scheduled follow-up appointment that was not attended, this was approximately one percent (n = 154).

### Patient demographics

**Figure 1:**
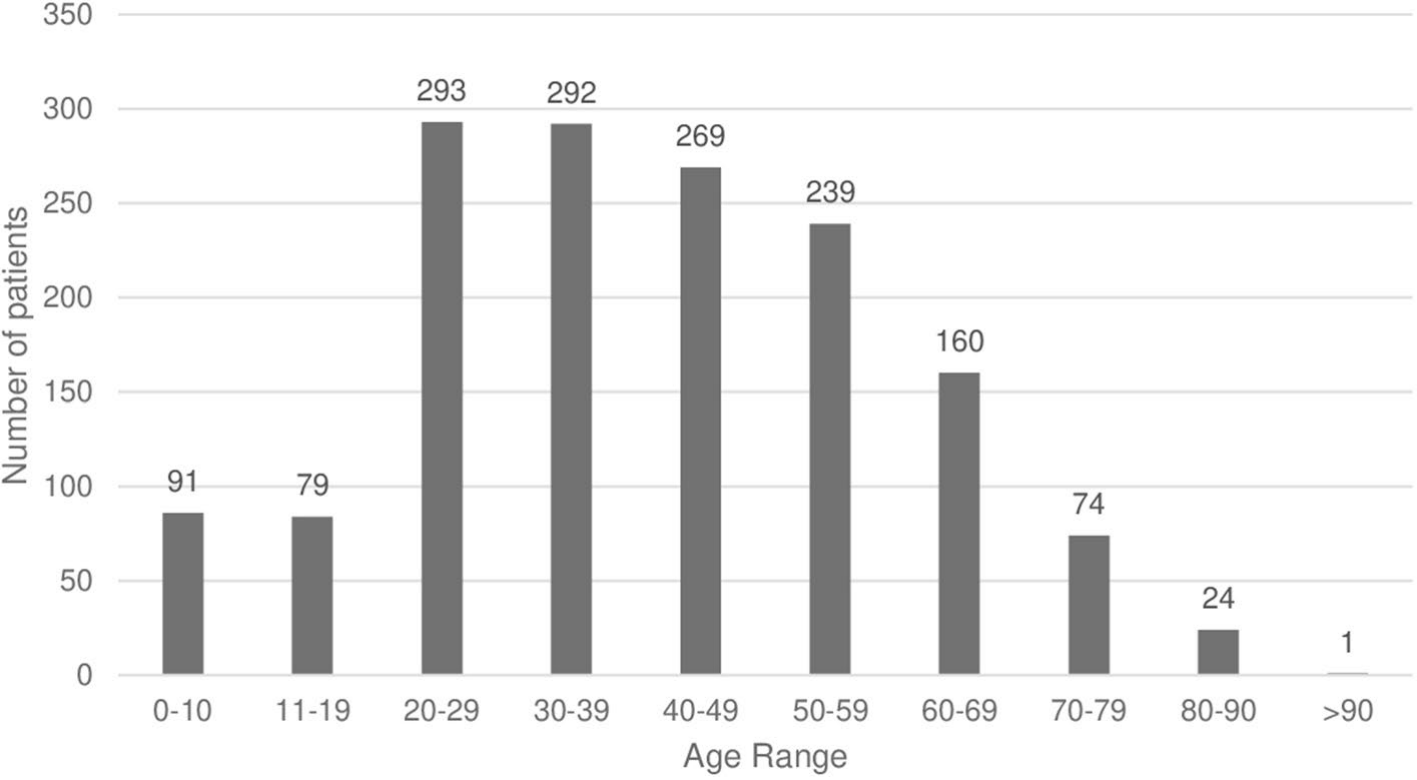
Age distribution of patients presenting with chemical eye injuries.

Based on the population served, the estimated incidence of significant chemical injuries was 9.5 ± 4.3 per 100,000 people per year. A male preponderance was observed, with 984 (65%) male and 537 (35%) female patients. The most common ethnicity was New Zealand European 677 (45%) followed by Indian 131 (9%). The mean age was 40.6 ± 18.8 years with a median age of 40 (interquartile range 27, range 91), further demonstrated in Fig. 1. Childhood data was skewed towards older adolescents with the majority of cases being in those 16–18 years old as demonstrated in Table 1.

**Table 1:** Age distribution of children presenting with chemical eye injuries.

< 1	35
1–5	42
6–10	14
11–15	29
16–18	43
Total	163

The frequency of accidents increased slightly during November and December but did not demonstrate seasonality. The distribution of injuries was equal between right 38% and left eyes 36%, with 26% of patients sustaining a bilateral injury.

### Mechanism of injury

The majority of injuries occurred at home (62%), followed by work (32%), recreation sites (5%) or in a medical environment (such as iatrogenic injuries during another surgical procedure) (1%). Half of severe injuries occurred at work which was much higher than in mild injuries (32%), however, this was not statistically significant (*p* = 0.231). The most frequent activity that resulted in injury was cleaning, both domestic and industrial (39%), followed by trade work (18%), then recreation (8%). Trade work encompassed a variety of activities including construction, electrical work, plumbing, mechanics and fixing industrial appliances. Recreation included all leisure activities and home-based maintenance activities. Cosmetic (4%) consisted mostly of eyebrow/eyelash treatments, hair dying and tattooing. Contact lens-related (2%) refers to patients putting their contact lenses in a solution and then not washing them sufficiently before reinserting or placing them in the wrong solution. Misidentification (2%) refers to patients putting a product in their eye, incorrectly thinking it was their eye medication. Assault/self-inflicted (1%) included intentional self-harm, and alleged assault. The mechanisms of injury are highlighted in Table 2.

**Table 2:** Activity at time of chemical injury.

Activity	Number of injuries (N = 1522)
Cleaning	597 (39%)
Tradeswork	277 (18%)
Recreation/DIY	127 (8%)
Gardening	121(8%)
Working with chemicals	84(6%)
Personal hygiene	74 (5%)
Cooking/Eating	66(4%)
Cosmetic	54 (4%)
Misindentification	29 (2%)
Contact lens related	28 (2%)
School science experiment	22(1%)
Assault/self-inflicted (including children)	16(1%)
Unknown	16(1%)
Medical/iatrogenic	11(1%)

Approximately half of the injuries (n = 753) were caused by alkali substances, 19% (n = 295) by acidic substances and 30% (n = 469) by a chemical that could not be definitively classified as acidic or alkaline (such as alcohol or food). The most common type of causative product was industrial-grade chemicals (36%), including chemicals used in home maintenance activities such as degreasers and paint strippers, followed by household cleaning products (32%). The dataset also included 62 cases of combined chemical and thermal burns.

### Clinical characteristics

Of the 1522 patients with chemical injuries, 1440 had presenting best corrected visual acuity (BCVA) recorded and 1371 had discharge visual acuities recorded. Of the 82 patients where initial visual acuities were not recorded, 67 (81.7%) occurred in small children who were too young for a formal visual assessment. The vast majority of patients (82%, n = 1440) had a presenting visual acuity of ≥ 6/12 (LogMar 0.30), and 10% (n = 154) were < 6/12 but > 6/30, of which 95 cases were directly attributable to the injury. There were fewer cases of more severe visual impairment with 4% (n = 57) between 6/30 and > 6/60, with 31 cases directly attributable to the injury. However, 3% were (n = 45) ≤ 6/60 or worse, with 30 cases directly attributable to the injury. Cases were defined as attributable to the injury if there was a difference between admission and discharge visual acuity. The missing discharge visual acuities were typically due to patients being lost to follow-up (1%) or transferred to other centres (0.5%) with no record of final visual acuities available. In over 80% of cases, there was no difference between visual acuity on admission compared to discharge.

Injury severity was categorised using the Dua classification for chemical injuries which accounts for the degree of limbal ischaemia and conjunctival involvement^9^. The vast majority of cases were mild; Grade I 1490 (98%) and Grade II 22 (1.5%). There were 5 moderate injuries Grade III (0.3%), and 5 severe injuries; Grade IV 1 (0.07%), while only 4 (0.3%) were Dua Grade VI. There were no cases of Dua Grade V injuries. Around one-third of patients (34.9%) referred with chemical injury had no sign of ocular injury at the time of presentation and another third (34.2%) had only punctate epithelial erosions of the cornea.

An epithelial defect was noted in 409 cases (26.9%). Dua Grade I cases were typically reviewed one week following the initial presentation and of those who attended scheduled follow-ups the vast majority (95%, n = 359) of epithelial defects had healed. All epithelial defects in these mild injuries that attended follow-up were healed by day 30. Of the Dua Grade II injuries, 80% (n = 16) achieved epithelisation at 7 days (all healed by 51 days), and of the Dua Grade III injuries, 60% (n = 3) of cases achieved epithelisation at 7 days and all healed by 17 days. The only Dua Grade IV injury took approximately 90 days for epithelisation. Dua Grade VI injuries took the longest to heal with one persistent epithelial defect still unhealed and a mean duration of 126 days to epithelisation in the other 3 cases.

For mild injuries (N = 1512) (Dua Grade I and II), the median admission visual acuity was 6/6 or LogMar 0.0 (range LogMar 3.0) and the median discharge visual acuity was also 6/6 or LogMar 0.0 (range LogMar 3.0). All mild injuries with vision 6/18 or worse at discharge were secondary to longstanding established visual impairment from other causes and had no change between admission and discharge visual acuity. For moderate injuries (N = 5) (Dua Grade III), the median visual acuity at admission was slightly worse at 6/7.5 (range LogMar 3.0) but the median discharge visual acuity was also 6/6 (range LogMar 3.0).

For severe injuries (Dua Grade IV, V and VI), the admission visual acuities were significantly worse with the majority (80%, n = 4) being hand movements (LogMar 2.4) or count fingers (LogMar 2.1) vision. By the time of the last follow-up, half of the patients still had at least one eye with 6/60 vision (LogMar 1.0) or worse. A severe injury was associated with a higher risk of longstanding visual impairment secondary to chemical injury when compared to mild injuries (*p* = 0.003).

These severe cases are discussed in more detail in the case series below.

### Management

Management of chemical injuries was dependent on severity. Irrigation duration and quality was variable and poorly documented. All injuries were irrigated at some stage, usually at the scene as well as well as either in the emergency department or in acute eye clinic. However, some injuries had significant delays in presentation and often were not irrigated at the time of injury. There was a higher proportion of moderate (Dua grade III) or severe (Dua grades IV–VI) injuries among those with no documentation of receiving ocular irrigation at the scene (90.0%) compared to those who did receive irrigation (10.0%) (*p* = 0.0001). In mild (Dua grade I–II) injuries almost all (98.2%) were irrigated at the scene. Lack of irrigation at the scene was associated with a statistically significant increased risk of longstanding visual impairment post-injury (*p* = 0.0001). Within our centre, a standardised, previously published, acute management protocol was followed upon arrival of the injured patient^10^.

Following acute management, those patients with mild injuries were typically treated with topical antibiotics (78%), preservative-free lubricating drops (77%) and occasionally topical steroids (16%). The treatment regimens received are further outlined in Table 3.

**Table 3:** Treatment of chemical injuries stratified by injury severity.

Treatment	Mild (DUA I and DUA II)	Moderate (DUA III)	Severe (DUA IV + V + VI)	Number of patients	Percentage
	(n = 1512)	(n = 5)	(n = 5)		
Topical antibiotic + PFAT + /− cyclo	620 (41.0%)	1 (20%)	0	621	40.80%
PFAT	270 (17.9%)	0	0	270	17.70%
Topical antibiotic + PFAT + Topical steroid + /− cyclo	301 (19.9%)	0	0	301	19.80%
Topical antibiotic	162 (10.7%)	0	0	162	10.60%
Intensive protocol: topical antibiotic + PFAT + topical citrate/ascorbate + oral vit C + oral tetracycline + topical steroid + cyclo	65 (4.3%)	4 (80%)	5 (100%)	74	4.90%
Nil	67 (4.4%)	0	0	67	4.40%
Topical steroid	24 (1.6%)	0	0	24	1.60%
Topical antibiotic + PFAT + anti-OHTN	3 (0.2%)	0	0	3	0.20%
Total				1522	

PFAT, preservative free artificial tears; anti-OHTN, anti-ocular hypertensive agent; vit C, vitamin C; oral tetracycline; cyclo, cycloplegic agent.

Patients with moderate (Dua Grade III) and severe (Dua Grade IV–VI) injuries were routinely admitted to the hospital for intensive treatment. Forty-seven (3%) patients required hospital admission. The management protocol is outlined in Table 3 and consists of preservative-free antibiotics, preservative lubricating drops, preservative-free topical steroids, a preservative-free cycloplegic, topical citrate 10% and ascorbate 10%, oral doxycycline and oral Vitamin C. Five patients required surgical management. In the acute phase, all five of these patients underwent amniotic membrane transplantation with 3 achieving epithelialisation. Two had chronic limbal stem cell failure and required limbal stem cell transplantation and penetrating keratoplasty.

### Illustrative cases of severe ocular chemical injuries

Four patients with severe ocular chemical burns (Dua Grade VI) highlight the complexities of managing these injuries. Of these four cases, three had both eyes affected and three required surgical management. None of these individuals were wearing eye protection at the time of injury. Two cases had at least one eye < 6/60 vision at the latest follow-up, with one patient having both eyes < 6/60.

**Figure 2:**
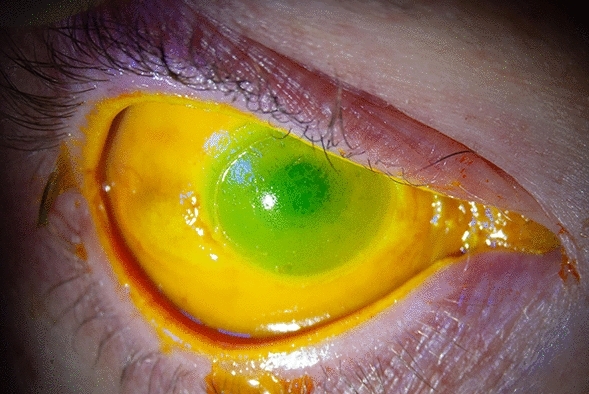
The right eye of case 2 on presentation.

Case 1. A 45-year-old male sustained bilateral injuries while attempting to unblock a pipe on a fishing boat with industrial chemicals. The right (OD) and left (OS) eyes had 360 and 180 degrees of limbal ischaemia, respectively. On presentation, BCVA was hand movements in OD and count fingers OS. His right eye on presentation is shown in Fig. 2. He required hospitalisation and intensive management including six bilateral amniotic membrane grafts. He developed an opaque, vascularised cornea OD secondary to limbal stem cell failure with vision limited to hand movements. He eventually achieved epithelilisation with BCVA 6/9 OS. Unfortunately, the patient died from unrelated circumstances a year later.

Case 2. A 40-year-old male was involved in a chemical explosion in an unregistered laboratory and sustained bilateral ocular chemical injuries. On admission, he had 360 degrees of limbal ischaemia with count fingers vision in both eyes. Over three years, he has required 34 bilateral amniotic membrane transplants. He developed bilateral limbal stem cell failure and underwent three living-related donor limbal cell transplants in the left eye and two in the right eye. He subsequently underwent bilateral penetrating keratoplasty. Currently, his BCVA is 6/45 OD and 6/21 OS. This case required 55 operations, 4 weeks in hospital and > 70 follow-up appointments and requires ongoing monitoring, highlighting the significant personal and societal cost of severe ocular chemical burns.

Case 3. A 38-year-old male sustained bilateral ocular chemical injuries as a result of hot bleach. At the time of injury, he had 360° of limbal ischaemia OD and 20° of limbal ischaemia OS. His vision was hand movements in the OD and 6/24 OS. He underwent eight amniotic membrane transplants bilaterally. His right eye became phthisical and was eviscerated a year post-injury. He underwent a living-related donor limbal stem cell transplant OS and at the last review, the BCVA was 6/120.

Case 4. A 79-year-old male sustained a right ocular chemical injury while mixing chlorine. On admission, his visual acuity was 6/60 OD and he had 360° of limbal ischaemia. He was treated with intensive medical treatment in accordance with our protocol. The time to epithelilisation was 49 days, however, visual acuity improved to 6/9 OD on discharge.

## Discussion

The reported incidence of ocular chemical injuries varies globally. A study in the US estimated the incidence as 10.7 per 100,000 per year,^11^ while a study of 239 severe injuries based in Shanghai estimated prevalence at 1.58 per 100,000^1^ and a Swiss study estimated it to be as high as 50 per 100,000^5^. Our study incidence of 9.5 per 100,000 is, therefore, in keeping with other international datasets from urbanised countries, whereas the prevalence in developing countries may be even higher. A study of cocoa workers in Ghana reported an incidence of 200 per 100,000 chemical injuries^12^.

There appear to be geographic differences with respect to the setting where the injury occurred. The chemical injuries in our study occurred primarily in the home (62%), as did those in the UK^13^, in stark contrast to the Shanghai study which reported 78% of its injuries occurred in the workplace^1^. This may be in part due to the strict eye protection guidelines in the workplace enforced by New Zealand law^14^. This further demonstrates the significance of protective eyewear in the prevention of chemical eye injuries. Interestingly, of our four most severe cases, two occurred in the workplace but all four were not wearing safety glasses at the time of injury.

Despite variable initial management at the setting where the injury occurred, 69% of patients presented with no discernible injury or only punctate epithelial changes at the time of review. The relatively low incidence of severe injuries and preponderance of good visual outcomes is also shared by other similar studies. A Swiss study of 163 patients found only 2% of patients had severe injuries, a UK study found 4% of injuries were Roper-Hall grade IV and 96% of injuries were treated only with topical medication, while a study from the US found that 74% of injuries had an admission visual acuity of 20/40 (6/12) or better^5,13,15^. This highlights the broad range of severity of chemical injuries presenting to eye emergency services, both locally and internationally. Although the majority are mild, a small but significant number of potentially blinding cases occur, and these cases ultimately carry significant attendant burdens for both the individual and health care services.

There was considerable variation in the prescribing patterns for the treatment of mild chemical injuries. The most common treatment regimen was a combination of topical antibiotics and preservative-free artificial tears (38.3%), followed by preservative-free artificial tears only (17.9%), and a combination of topical antibiotics, preservative-free artificial tears, and topical steroids (16.9%). A minority of patients (4.3%) received intensive treatment despite only having mild injuries. Irrespective of the treatment regimen, mild ocular chemical injuries healed well, and nearly all eyes (99%) achieved complete re-epithelisation within one week. A previous retrospective study by Brodovsky et al. suggested that topical steroids in the context of mild alkali injuries may delay re-epithelisation^16^ likely due to drop toxicity and the corticosteroid suppression of corneal healing. We therefore recommend that clinicians consider the therapeutic benefit to risk ratio in the management of these mild ocular chemical injuries and prescribe accordingly.

Brodovsky also demonstrated that the use of an intensive treatment protocol in moderate injuries showed a trend towards more rapid healing time and improved final visual outcomes^16^. In the current study, all moderate injuries except one were treated with a comparable intensive treatment regimen. The mean time to re-epithelisation in this group was 8 days, whilst Brodovsky’s reported 10 days,^16^ suggesting this is an effective treatment of moderate injuries. While historically there has been concern regarding the prolonged use of topical steroids, our study corroborates the work of other researchers in demonstrating that topical steroids used in conjunction with topical ascorbate/citrate are not associated with significant corneal stromolysis^16^.

The poor prognosis of severe ocular chemical burns is well-established in the literature and is echoed in the present study. Notably, several studies of severe chemical injuries typically report poor visual outcomes and persistent epithelial defects, despite appropriate intensive treatment regimens that may ultimately require surgical intervention^1,13,16,17^. It can be difficult to ascertain the strengths of the treatment regimen for such severe injuries due to smaller patient numbers, but in the current study, more than half of the eyes (Dua grade IV–VI, N = 5) retained vision > 6/60 on discharge, and all but one epithelial defect eventually healed, suggesting that intensive intervention is beneficial even in eyes with a poor prognosis. These severe injuries occur primarily in young working-age men and present a significant economic burden on the health service, with an estimated cost of eye trauma to New Zealand at around 3 million NZD per year^14^. However, this number may be greater when we consider that these individuals are often out of the workforce for several years, and may never be able to return to their previous vocation. The most severe injuries occurred in the context of no eye protection and poor initial irrigation. Hence, prevention of these injuries with adequate eye protection and high-quality first aid is paramount.

There are some notable limitations of this study. Because it is possible that not all patients present for emergency eye care or are referred to the emergency eye clinic in Auckland, it is possible the calculated incidence rate is a low estimate. However, it is presumed that this study captured the majority or all of those with severe injuries, as they would be less likely to not seek care. In addition, it is possible that the electronic data search could have missed some cases that were not correctly documented or coded, also resulting in an underestimation of the calculated incidence rate. It was challenging to accurately estimate the burden of visual impairment due to confounding from long-standing poor vision and a lack of discharge visual acuity. Discharge visual acuity data was limited as many mild cases were not required to present for follow-up, and some severe cases returned to their home domicile for follow-up. Our analysis was limited by the quality of documentation, and analysis based on eyes rather than patients may have provided more detailed information in the case of bilateral chemical injuries. Also, documentation regarding eye protection at the scene of the injury was not complete for all patients such that no statistical evaluation of this potential risk factor was performed.

To our knowledge, this study represents the largest study of ocular chemical injuries conducted in Oceania. While there are limitations inherently associated with a retrospective review, the study provides insights into the management and outcomes of patients with ocular chemical injuries. Fortunately, the majority of injuries were mild and healed well. Moderate injuries responded well to the intensive treatment protocol, but due to the small number of patients and lack of a control group, it is not possible to draw definitive conclusions regarding its efficacy. Although rare, severe injuries carry a poor prognosis despite maximal care. Even a small number of severe chemical injuries are associated with a substantial economic burden. Improving patient awareness around ocular safety is essential for the prevention of profound chemical injuries.

## Data Availability

The datasets generated and/or analysed during the current study are not publicly available due to ethics and confidentiality guidelines held by the University of Auckland but are available from the corresponding author upon reasonable request.
